# PKPD modeling of acquired resistance to anti-cancer drug treatment

**DOI:** 10.1007/s10928-017-9553-x

**Published:** 2017-10-31

**Authors:** Miro J. Eigenmann, Nicolas Frances, Thierry Lavé, Antje-Christine Walz

**Affiliations:** 1 Roche Pharma Research and Early Development, Quantitative Systems Pharmacology, Hoffmann-La Roche Ltd., Grenzacherstrasse, 124, 4070 Basel, Switzerland; 2 Pharmaceutical Sciences Roche Innovation Centre Basel, Hoffmann-La Roche Ltd., Basel, Switzerland

**Keywords:** Acquired resistance, PKPD model, Simulation, Tumor growth inhibition model, Tyrosine kinase inhibitor, Patient-derived tumor xenograft mice

## Abstract

Non-small cell lung cancer (NSCLC) patients greatly benefit from the treatment with tyrosine kinase inhibitors (TKIs) targeting the epidermal growth factor receptor (EGFR). However, emergence of acquired resistance inevitable occurs after long-term treatment in most patients and limits clinical improvement. In the present study, resistance to drug treatment in patient-derived NSCLC xenograft mice was assessed and modeling and simulation was applied to understand the dynamics of drug resistance as a basis to explore more beneficial drug regimen. Two semi-mechanistic models were fitted to tumor growth inhibition profiles during and after treatment with erlotinib or gefitinib. The base model proposes that as a result of drug treatment, tumor cells stop proliferating and undergo several stages of damage before they eventually die. The acquired resistance model adds a resistance term to the base model which assumes that resistant cells are emerging from the pool of damaged tumor cells. As a result, tumor cells sensitive to drug treatment will either die or be converted to a drug resistant cell population which is proliferating at a slower growth rate as compared to the sensitive cells. The observed tumor growth profiles were better described by the resistance model and emergence of resistance was concluded. In simulation studies, the selection of resistant cells was explored as well as the time-variant fraction of resistant over sensitive cells. The proposed model provides insight into the dynamic processes of emerging resistance. It predicts tumor regrowth during treatment driven by the selection of resistant cells and explains why faster tumor regrowth may occur after discontinuation of TKI treatment. Finally, it is shown how the semi-mechanistic model can be used to explore different scenarios and to identify optimal treatment schedules in clinical trials.

**Electronic supplementary material:**

The online version of this article (10.1007/s10928-017-9553-x) contains supplementary material, which is available to authorized users.

## Introduction

Targeted therapies such as tyrosine kinase inhibitors (TKIs) are widely used for cancer treatment. First generation of TKI includes erlotinib and gefitinib, two small molecule drugs with similar chemical structures which have been approved for the treatment of NSCLC [1–3] and subsequently of various solid epidermoid cancers [4, 5]. Both molecules target the ATP binding site of EGFR and inhibit EGFR-induced activation of downstream signaling [6]. It has been reported that long term treatment with these EGFR-TKI drugs eventually leads to emergence of resistance in most patients [1, 7, 8]. Two main type of resistance have been described. Primary resistance, referring to patients who are not responsive to the drug treatment and secondary or acquired resistance, which is usually defined as progression of disease after an initial period of clinical benefit while the patient is still under treatment [9]. Underlying mechanisms of resistance towards TKI drugs have been thoroughly discussed in literature [8–14]. The most frequently described molecular mechanism of acquired resistance occurs in exon 20 (T790 M) [15–18]. This change leads to altered binding of the drug within the ATP pocket [19]. Tumors with T790 M mutations were found to have a slower, less aggressive course of tumor progression [16, 20–22]. Despite initial responses, drug effect drops and patients eventually progress due to acquired resistance. In these cases, TKI treatment gets commonly withdrawn and second line treatment is applied. However, some reports mention that even though resistance to the TKI treatment was identified, a discontinuation of the treatment leads in some cases to a subsequent disease flare [10, 15, 20]. Strategies to overcome resistance involve combination therapies [23], mutant selective treatments and altering conventional treatment schedules [22, 24–26]. Mathematical modeling has been applied in numerous cases to investigate emergence of resistance and exploring possibilities to overcome it [22, 27–30].

We recently conducted a translational PKPD study based on in vitro and in vivo experiments to quantitatively compare erlotinib and gefitinib and to retrospectively predict efficacious dose in humans [31]. The two compounds were selected based on the strong tumor uptake differences observed for erlotinib and gefitinib despite similar molecular specificity. It was demonstrated that active drug exposure at target site is higher for erlotinib despite having lower total tumor to plasma ratios. Furthermore; the model-based dose prediction matched the recommended clinical doses well. However, model misspecification was apparent in the high dose group which was not relevant to the address the question of our previous work. It was speculated that this was due to emergence of resistance and therefore, these data were further explored in the present study to investigate this in patient-derived NSCLC xenografts obtained by direct implants of surgically resected tumors in mice. This animal model maintains morphological similarities, tumor heterogeneity and recapitulates molecular profiling of the original tumors [31]. The goal of this work was to test if acquired resistance to treatment with erlotinib and gefitinib can be observed in preclinical studies and to simulate the dynamics of emergent resistance. Applying parsimonious principles, we compared model performance of two semi-mechanistic PK/PD models with and without acquired resistance. This allowed confirming or excluding emergence of resistance in xenograft mice. With the final model, simulation studies were conducted and the selection of resistant cells as well as time-variant fraction of resistant to sensitive cells was predicted. The proposed model provides insight into the dynamic processes of emerging resistance and allows exploration of dosing regimen.

### Theoretical

#### PK model

An oral, 1-compartment model with first order absorption and first order elimination, was fitted to the PK data from the short and the long term experiments.

The PK model can be described with the following equations:

1 $$\frac{{dA_{d} }}{dt} = - \,k_{a} \times A_{d} \quad A_{d} \left( 0 \right) = \,D$$

2 $$\frac{{dA_{p} }}{dt} = k_{a} \times A_{d} - k_{e} \times A_{p} \quad Ap\left( 0 \right) = 0$$

3 $$C_{p} = \frac{{A_{p} }}{V - (D * I)}$$

where $A_{d}$ corresponds to the quantity of drug in the depot compartment, $Ap$ [µg] to the amount of drug in plasma, $C_{p}$[µg/L] to the plasma concentration and $D$ [mg/kg] is the weight normalized dose. $V$ [L] represents apparent volume of distribution, $k_{a}$ [1/d] corresponds the absorption rate and $k_{e}$ [1/d] to the elimination rate. For gefitinib, a dose-dependent decrease in volume of distribution was observed and captured with the gefitinib-specific parameter *I,* while it was fixed to 0 for erlotinib. Individual PK parameters estimates are later fixed in the PKPD models.

#### TGI base model

Tumor volume (*TV*) is composed of dividing cells (*S*) and damaged cells due to drug treatment (T1–T3). Unperturbed tumor growth is described by a nonlinear growth model. This model assumes a continuous switch from exponential to linear growth behavior as the tumor volume increases [32]. The drug effect is delayed relative to the drug exposure in plasma and captured by a signal transduction model as described by Simeoni and colleagues [33]. We estimated the drug effect via a killing function involving a direct and linear effect model [34]. It is described with the following equations:

4 $${\text{TV = }}\,{\text{S + T1 + T2 + T3}}$$

5 $$\frac{dS}{dt} = \frac{{2 \times \lambda_{0} \times \lambda_{1} \times S}}{{2 \times \lambda_{0} \times S + \lambda_{1} }} - k_{2} \times C_{p} \times S\quad S\left( 0 \right) = TV_{0}$$

6 $$\frac{dT1}{dt} = k_{2} \times S \times C_{p} - T1 \times k_{1} \quad T1\left( 0 \right) = 0$$

7 $$\frac{dT2}{dt} = k_{1} \times (T1 - T2)\quad T2\left( 0 \right) = 0$$

8 $$\frac{dT3}{dt} = k_{1} \times (T2 - T3)\quad T3\left( 0 \right) = 0$$

where $TV_{0}$ is the tumor volume at the start of the study, $\lambda_{0}$ and $\lambda_{1}$ are the exponential and linear growth parameters, $k_{1}$ is the rate accounting for the delayed effect and $k_{2}$ represents the potency parameter of the compound.

#### Acquired resistance model

**Fig. 1:**
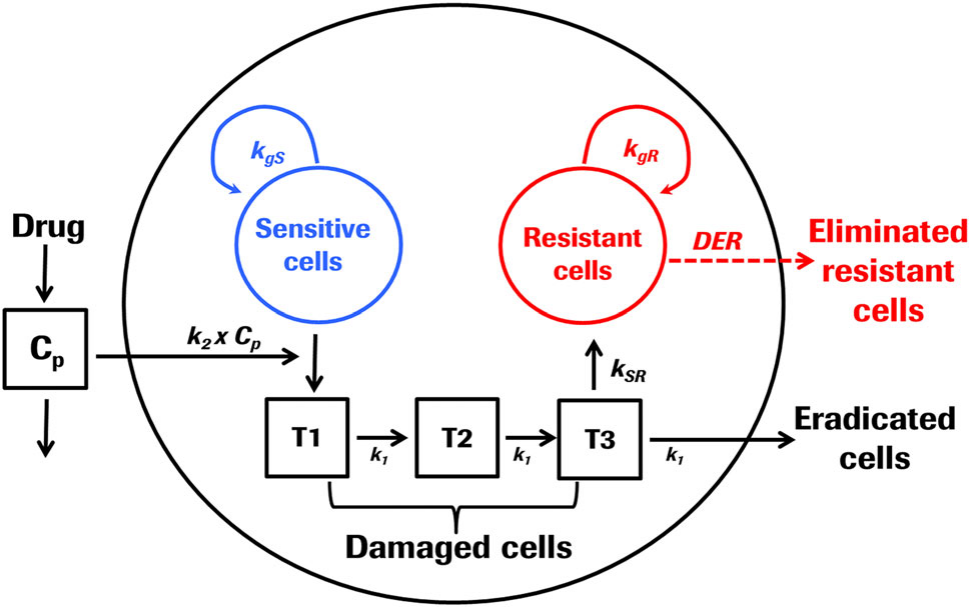
Model structure of acquired resistance. As a result of drug administration, tumor cells sensitive to drug treatment undergo several stages of damage (T1, T2, T3) before the eventually die or get converted to drug-resistant cells. Both, resistant and sensitive cells are proliferating. Total tumor comprises sensitive, damaged and resistant cells and fraction of resistant cells changes over time and as a function of drug treatment. If drug concentration is above a threshold concentration Cth, the resistant cells are eliminated by the drug action. It is of note however that drug effect on resistant cells (*DER*) with concentrations above threshold was used for simulations only as no such effect could be identified during model parameter estimation

The base model is extended to capture acquired resistance by introducing a compartment *R* representing the resistant cell population which emerges upon drug treatment (Fig. 1). As a result of drug treatment, tumor cells sensitive to drug treatment undergo several stages of damage (*T1*–*T3*), which are either killed or converted to a drug resistant cell population with *kSR* describing the first order conversion process as proposed by Li and colleagues [30]. The conversion is integrated into the model as a delayed process, consistent with the delayed drug effect. For the resistant cell populations, the same structural model was assumed for tumor growth but with distinct linear and exponential growth rates. The parameter *β* denotes the ratio of growth rate of resistant versus sensitive cells (Eq. 15). Total tumor (*TV*) is the sum of sensitive (*S*), damaged (*T1*–*T3*) and resistant (*R*) cells. A schematic representation of the structural model is provided in Fig. 1 and is described by the following equations:

9 $$TV = S + T1 + T2 + T3 + R$$

10 $$\frac{dS}{dt} = \frac{{2 \times \lambda_{0} \times \lambda_{1} \times S}}{{2 \times \lambda_{0} \times S + \lambda_{1} }} - k_{2} \times C_{p} \times S\quad S\left( 0 \right) = TV_{0}$$

11 $$\frac{dT1}{dt} = k_{2} \times S \times C_{p} - T1 \times k_{1} \quad T1\left( 0 \right) = 0$$

12 $$\frac{dT2}{dt} = k_{1} \times (T1 - T2)\quad T2\left( 0 \right) = 0$$

13 $$\frac{dT3}{dt} = k_{1} \times \left( {T2 - T3} \right) - kSR \times T3\quad T3\left( 0 \right) = 0$$

14 $$\frac{dR}{dt} = \frac{{2 \times \lambda_{0R} \times \lambda_{1R} \times R}}{{2 \times \lambda_{0R} \times R + \lambda_{1R} }} + kSR \times T3 - {\text{DER}}\quad R\left( 0 \right) = 0$$

And with the following relationship between the growth rates of sensitive and resistant cells:

15 $$\beta = \frac{{\lambda_{0R} }}{{\lambda_{0} }} = \frac{{\lambda_{1R} }}{{\lambda_{1} }}$$

where *τ* denotes the average time it takes for tumor cell to be eradicated or to convert to resistant cells given that $kSR$ ≪ $k_{1}$ and $kSR$ can be neglected. This secondary parameter is derived from the following equation:

16 $$\tau\, = \frac{3}{{k_{1} }}$$

Assuming a drug effect on the resistant cells (DER) when plasma concentration (C_P2_) is above the threshold concentration C_th_

17 $$DER = k_{2R} \times C_{p2} \quad if\,C_{p2} > C_{th} \,else\,{\text{DER}} = 0$$

The threshold concentration *C*_*th*_ was fixed based on an in vitro study [20] reporting killing of resistant cells when exceeding the threshold concentration (*C*_*th_in_nvitro*_) of 1 μM of erlotinib (MW = 393.4 g/mol) and converted to a total plasma threshold concentration (*C*_*th*_) of 7150 μg/mL by correcting for fraction unbound ($$fu)$$ of 0.055 [35].

18 $$C_{th} = \frac{{C_{th\_in\_vitro} }}{{f_{u} }}$$

## Methods

### In vivo tumor growth inhibition study

Tumor growth inhibition (TGI) studies were conducted in primary patient NSCLC tumor (LXF A677) bearing mice receiving erlotinib or gefitinib treatment. Experimental details have been previously described elsewhere [31]. In summary, the mice were randomized into 7 groups with 8 animals per group. Each group received either low, mid or high dose of erlotinib or gefitinib or vehicle only (control group). The drugs were administered orally once per day for 14 days. Tumor volume was monitored by caliper measurements over 30 days and sparse PK samples were collected. The detection limit for tumor volume was 5 mm^3^. The experimental study design is summarized in table S1, supplementary material. An overview of missing data due to early determination and data below limit of quantification is summarized in supplementary table S2.

### Parameter estimation

PKPD parameters were estimated using a population approach with Monolix Version 4.2.2. (Lixoft), allowing for estimation of fixed and random effects for each model parameter and a residual error in one step [36]. Residual errors for the PK were assumed to be proportional to predicted concentrations. For the PD, a combined error model was selected. Diagnostic plots were inspected to select the appropriate error model.

PK parameters estimated in previous report [31] were used in the present study. The individual PK parameter estimates were fixed and served as input in the PKPD modeling.

PK parameters which were estimated and reported in a previous publication [31] were used in the current study. The individual PK parameter estimates were fixed and served as input in the PKPD modeling. PD parameters were estimated simultaneously by combining erlotinib and gefitinib datasets while assuming the same tumor growth rates of sensitive and resistant cells, delay parameter and transfer rate to resistant cells. To allow a precise and stable estimation of fixed effects, random effects were fixed to low values if necessary. Model evaluation and selection was based on model convergence, precision of the parameter estimates, fitting criteria (Akaike Information Criteria (AIC)) and visual inspection of diagnostic tools such as visual predictive checks, residuals and observed versus predicted plots.

### Covariates

Covariates were tested for both PKPD models to test if parameters change with dose. Based on the results (data not shown) high dose treatment (100 mg/kg) was identified as covariate for the transformation rate *kSR* in the resistance model (Eqs. 9–14) and, in case of erlotinib, for the efficacy parameter *k2*. The high dose group was assessed as categorical covariate in Monolix. Fixed effect and random effect for the respective parameters are estimated for each defined category (here low/mid vs. high dose) and the statistical significance of differences between corresponding estimates is evaluated and the corresponding *p* value indicated. No PD covariates were identifiable and significant (*p* value below 0.05) in case of the base TGI model (Eqs. 4–8). A dose dependent change in the volume of distribution was observed for gefitinib and computed by the term $V - \left( {D*I} \right)$ (Eq. 3) as reported in [31]. *I* was estimated for gefitinib and fixed to 0 for erlotinib (no dose effect observed).

### Simulation studies

Simulation studies were performed in Berkeley Madonna v8.3.18. The aim of these simulation studies was to generate insights into dynamic changes of heterogeneous tumors under drug treatment and to explore in silico other treatment options in the preclinical as well as in the clinical setting. The model codes are presented in supplementary material (S3–S5). In order to compare dosing schedules in mice, inter-individual variability was included on the estimated model parameters (S4), 250 individual profiles were simulated based on random sampling from the respective parameter distribution space. The mean, the 5 and 95% confidence interval (*CI*) of the mean for these profiles and additionally derived metrics were calculated:

19 $$CI = \mu \pm 1.96* \frac{\sigma }{n}$$

where *μ* represents the mean, *σ* the standard deviation and *n* the number of samples. Additional metrics were quantified, minimal tumor volume achieved under the treatment schedule, time to progression and tumor burden over time (AUCE). The minimal tumor volume was derived from the simulation output. Time to progression was defined as timespan after treatment start until the tumor volume supersedes the tumor volume measured at treatment start. In order to show the impact of timing when the two dosing schedules are compared and the corresponding AUCE after 30 and after 60 days are reported.

## Results

### PKPD model reveals acquired resistance in patient derived xenograft mice

**Fig. 2:**
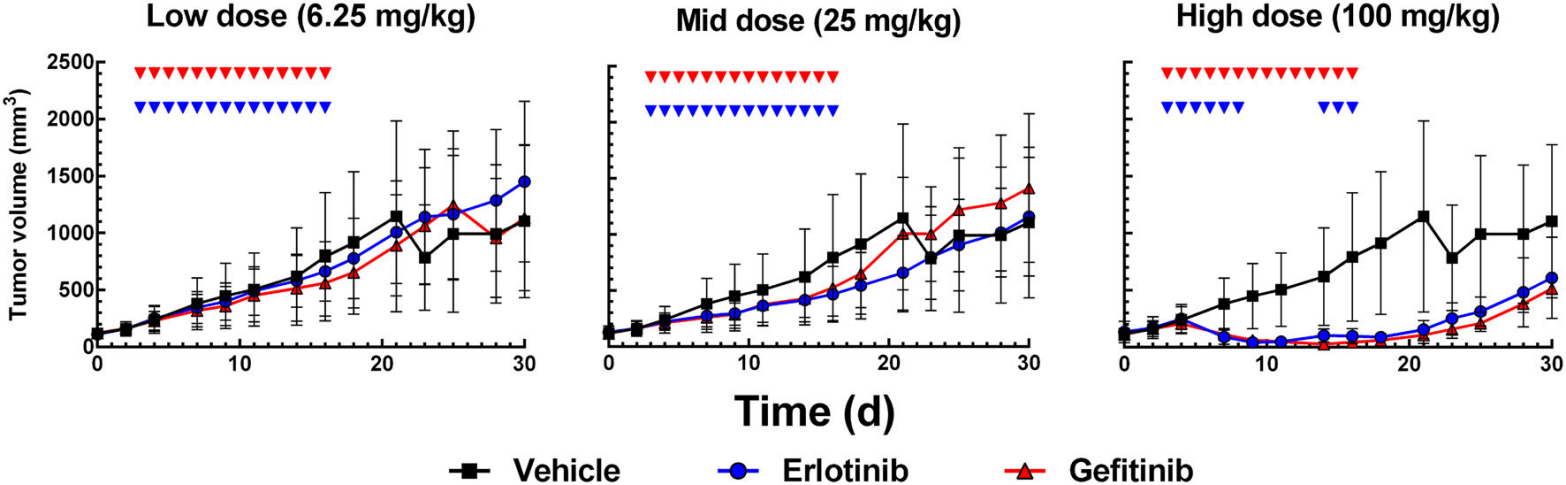
Tumor growth inhibiton profiles (mean data) per treatment and dose group. Gefitinib is shown in red and erlotinib in blue. Dosing schedule is indicated as small cubes above the curves

**Fig. 3:**
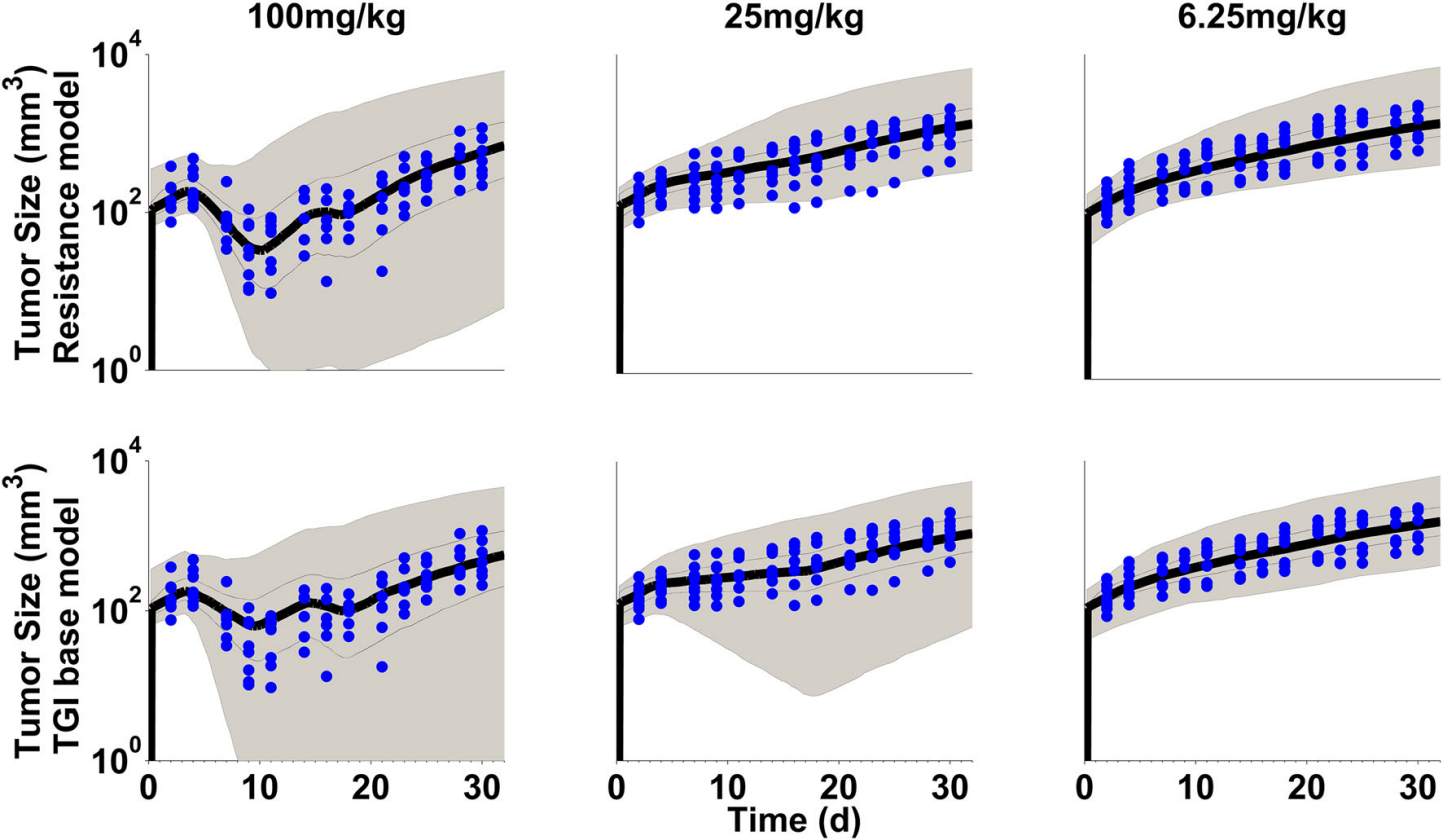
VPC with resistance model (upper panel) and with TGI base model (lower panel) after treatment with erlotinib at different dose levels

**Fig. 4:**
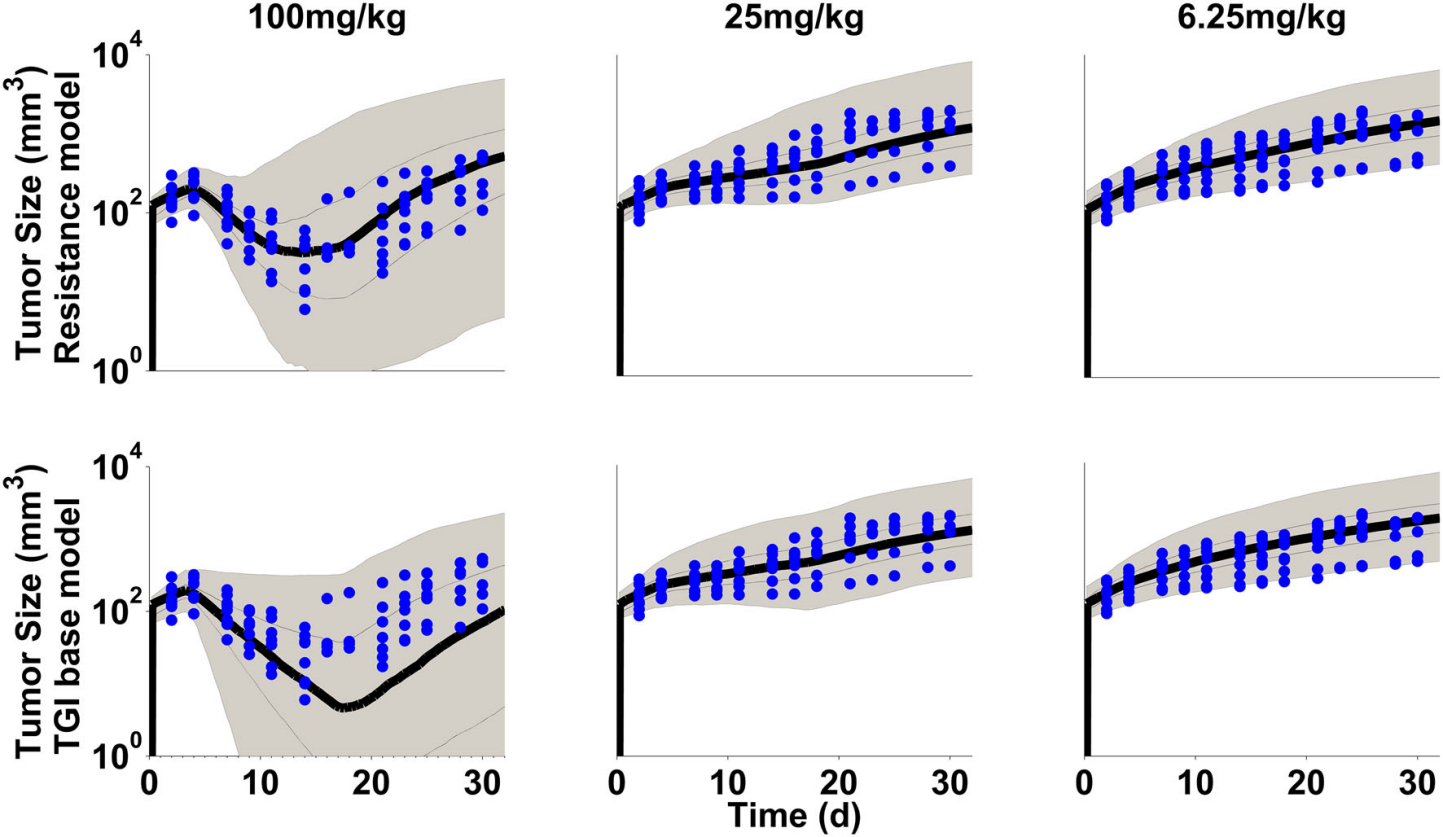
VPC with resistance model (upper panel) and with TGI base model (lower panel) after treatment with gefitinib at different dose levels

Data exploration (Fig. 2) shows dose dependent tumor growth inhibition in patient-derived NSCLC xenograft mice treated with erlotinib or gefitinib. Resistance to drug treatment was not obvious based on visual inspection of the mean data. However, some mice showed at the end of the treatment phase reduced response to drug treatment (Supplementary Fig S6) as indicated by visual inspection. In addition an unexplained bias occurred when the TGI data are described with a direct effect model [31]. Furthermore, it has been observed that in patients long term treatment inevitably leads to resistance [1]. These findings lead us to investigate potential emergence of resistance in this TGI study in xenograft mice. In order to confirm or exclude resistance to drug treatment, model performance of two semi-mechanistic PK/PD models was compared, with acquired resistance (Eqs. 9–14) and without resistance (Eqs. 4–8). Both models were fitted simultaneously to the individual data and the visual predictive checks are shown in Figs. 3 and 4. All individual observations are within the 90% prediction intervals and overall, the data are reasonably well described by both models. However, while the drug effect of the high dose group is over-predicted with gefitinib (Fig. 4, lower panel), it is under-predicted with erlotinib (Fig. 3, lower panel) with the base TGI model. This model bias disappears when the same data sets are fitted to the acquired resistance model (Figs. 3, 4, upper panels). The variability was over-predicted at the higher dose groups for erlotinib and gefitinib. The Ω-correlation matrix was evaluated and no correlation between parameters in its non-diagonal entries was observed (Supplementary Table S7). Table 1 summarizes the model performance metrics. While the resistance model has two additional parameters (n = 7), a lower Akaike information criterion score (AIC 7560) was achieved, indicating improved model performance of the resistance model as compared to the base TGI model (AIC 7720). In addition, the residual error (Table 1) is considerably decreased from 0.163 to 0.136 when adding a resistance term to the base TGI model which suggests a more precise data description by the acquired resistance model. In the present study, the total number of missing data was 7% as displayed in table S2 in supplementary material. The reason for missing data was either because the animals were sacrificed due to high tumor burden or due to occurrence of severe adverse events during the study. Another reason was that data points were below the detection limit (BLQ) of 6 mm^3^. It should be noted that all BLQ values were observed at the end of the treatment phase which was followed by observations during drug wash-out phase. In the final analysis, we handled BLQ data as missing information. During model building, BLQ values were treated as left-censored data, but it did not improve the model performance and did not reduce the bias.

**Table 1:** Model performance comparison of base TGI

Model evaluation	Base TGI model	Acquired resistance TGI model
# parameter	5	7
Residual error	0.163	0.136
AIC	7718	7550

*AIC* akaike information criterion

### TGI model with acquired resistance predicts slowly growing resistant cells

The final model parameter estimates for the resistance model are displayed in Table 2. All parameters were identifiable (Table 2) with standard errors below 25%. Random effects were high (0.468–0.689), but well estimated with low residual errors (below 25%). This high dispersion was expected given the high variability observed in the dataset. For both drugs, only one set of parameter was estimated for system related parameters, such as tumor growth rates $\lambda_{0}$ and $\lambda_{1}$. The drug-related parameter *k2*, which is a measure of drug potency was estimated separately for both drugs. As a result, for gefitinib a 2–7-fold higher *k2* value was observed (6.19 E−04 L/μg/d) as compared to high (of 3.15 E−04 L/μg/d) and mid/low dose (9.21 E−05 L/μg/d) of erlotinib, for which high dose was a covariate on this parameter. Given the similar mode of action of both drugs, it was assumed the transit rate (*k1*) and the conversion rate from damaged to resistant cells (*kSR*) are the same for both drugs and only one set of parameter was accurately estimated. The high dose group was identified as a covariate on the transformation parameter (*kSR*) resulting in fourfold lower rate in high dose (1.7 E−03 1/d) as compared to mid and low dose group (7.4 E−03 1/d). Residual effects for τ, *k2*-Gefitinib and *kSR* were not identifiable and therefore fixed to low values. The parameter *β*, denoting the ratio on growth rate of resistant versus sensitive cells (Eq. 15), was estimated to be 0.85. This result suggests slower growth of resistant cells.

**Table 2:** Final PKPD parameter estimates, resistance model

Parameter	Description	Fixed effect (RSE %)		Random effect (RSE %)	
		Erlotinib	Gefitinib	Erlotinib	Gefitinib
*ka* [1/d]	Absorption rate	55.0 (X)	55.0 (X)	–	–
*ke* [1/d]	Elimination rate	7.56 (10)	3.87 (10)	0.332 (39)	0.352 (20)
*V* [L]	Volume of distribution	0.127 (15)	1.40 (11)	0.251 (X)	0.0278 (X)
*I*	Factor denoting dose-dependent change in volume	0 (X)	0.00772 (22)	–	–
*λ*_*0*_ [1/d]	Exponential growth rate	0.217 (11)		0.487 (18)	
*λ*_*1*_ [mm^3^/d]	Linear growth rate	42.8 (10)		0.653 (11)	
*k1* [1/d]	Transit rate	1.52 (7)		0.2 (X)	
*β*	Ratio of growth rates of resistant versus sensitive cells	0.869 (18)		0.742 (18)	
*kSR* [1/d]	Transformation rate (damaged to resistant cells)	7.4 * 10^−3^ (8)		0.1 (X)	
*kSR* (high dose) [1/d]	Transformation rate (damaged to resistant cells), high dose group	1.7 * 10^−3^ (30) p = 2 * 10^−6^		–	
*k2* [L/μg/d]	Drug effect, slope parameter	9.21* 10^−5^ (23)	6.19 * 10^−4^ (8)	0.655 (23)	0.2 (X)
*k2* (high dose) [L/μg/d]	Drug effect, slope parameter, high dose	3.15 * 10^−4^ (17) p = 1.1 * 10^−5^	–	–	–
*τ (d)*	Average time for tumor eradication or emergence of resistance	1.97	–	–	–

(X) denotes parameter estimates that were fixed and therefore no relative standard error is reported. The *p* corresponds to p-values which indicates if the estimated *kSR*, and in case of erlotinib the estimated *k2*, is statistically different for the highest dose group (100 mg/kg) as compared to the mid and low dose treatment group (25 mg/kg and 5 mg/kg)

### Time-variant fraction of resistant cells of total tumor during and after treatment with erlotinib

**Fig. 5:**
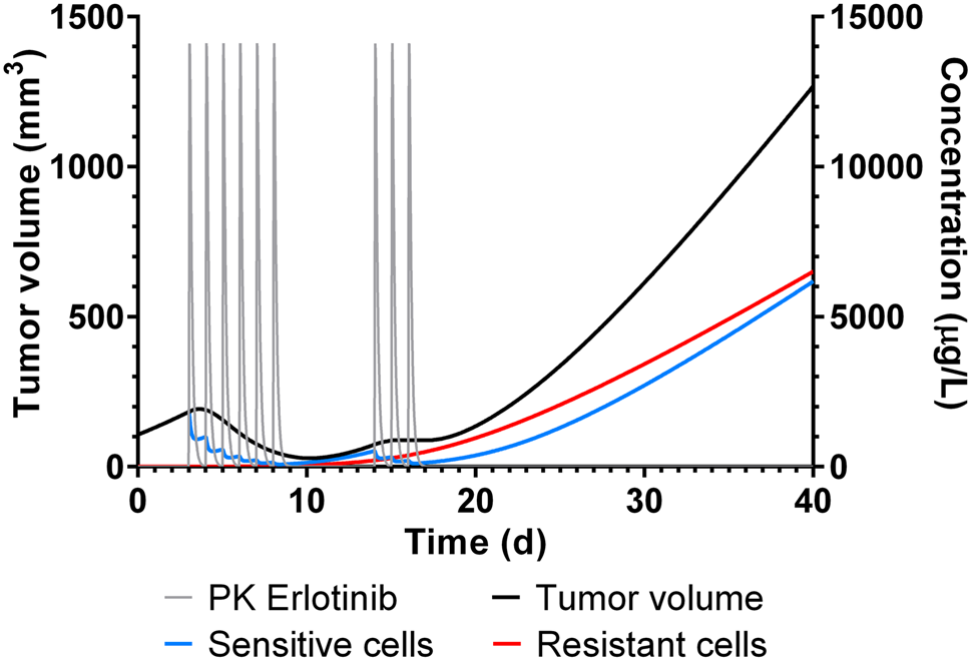
Simulation study in xenograft mice with 100 mg/kg erlotinib administered on day 3–8 and day 14–16. PK (grey), total tumor volume (black), as well as the tumor growth profiles of sensitive cells (green) and resistant (red) cells are shown

Since all parameters were well estimated, the model was applied to assess the dynamic processes of emerging resistance. For illustration, this in silico exploration was done for erlotinib only and the Berkeley Madonna code can be retrieved from Supplementary material (S3). First, the growth profile of total tumor was simulated as the sum of sensitive and resistant cells in xenograft mice (Fig. 5) treated with 100 mg/kg erlotinib, administered on day 3–8 and day 14–16 as tested experimentally. During the first treatment cycle, the total tumor decreases and enrichment of resistant cells remains negligible. However, during the second treatment cycle, total tumor volume remains constant due to two opposing effects, namely the reduction of sensitive and the enrichment of resistant cells.

**Fig. 6:**
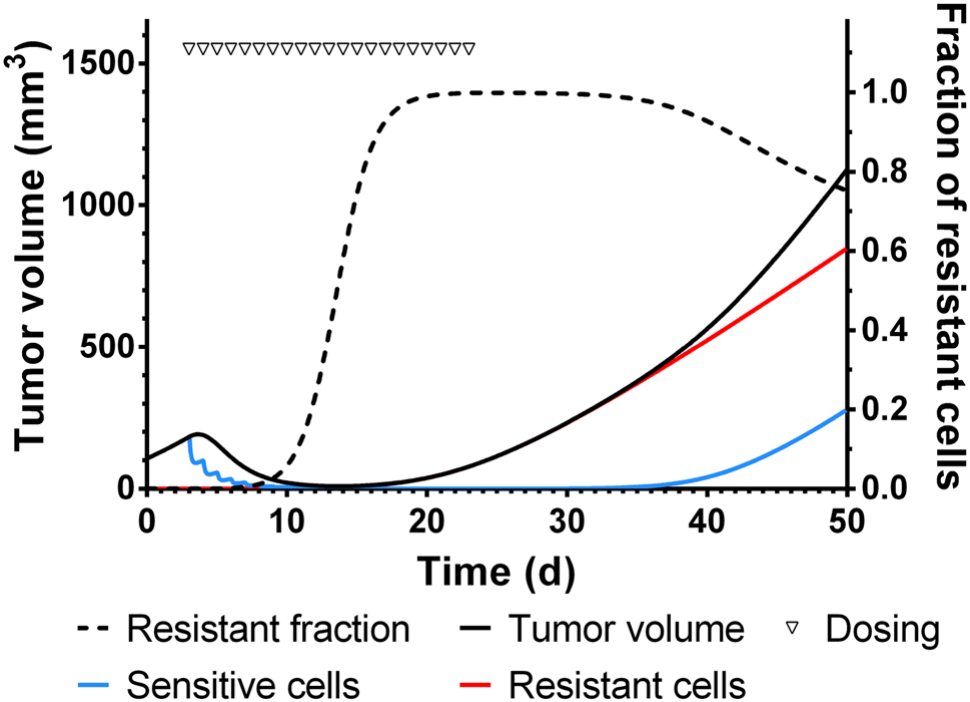
Simulation study in xenograft mice. Total tumor volume is the sum of sensitive and resistant cells which are enriched upon drug treatment. The fraction of resistant cells increases during drug treatment and decreases when treatment is stopped

Next, simulations were conducted to predict the outcome of a long term treatment study with 20 daily doses of 100 mg/kg erlotinib starting on day 3 followed by a wash-out phase of 27 days. The simulated profiles are presented in Fig. 6. At the start of the study, the tumor is composed of sensitive cells only and the profiles of total tumor (black) and sensitive cells (blue) superimpose. When high dose of drug is administered, the sensitive tumor cells decrease due to the drug induced cell killing. However, with drug treatment, the accumulation of resistant cells starts (Fig. 6, red line). As a result, total tumor shrinks and is now composed of sensitive, damaged and resistant cells. After initial response to drug treatment total tumor volume (Fig. 6, solid black line) and fraction of resistant cells (Fig. 6, dashed black line) increases, since the resistant cells are selected and assumed not to respond to drug treatment. Relapse of tumor occurs due to the slowly growing resistant tumor cells and when drug treatment is discontinued, the rate of tumor re-growth is markedly increased. This is explained by the fact that a small fraction of sensitive cells is starting to re-grow at their inherent higher growth rate. After removal of the drug, fraction of resistant cells decreases mainly because the sensitive cell population rapidly overgrows the resistant cells.

### Impact of dosing regimen on emergence of resistance

**Fig. 7:**
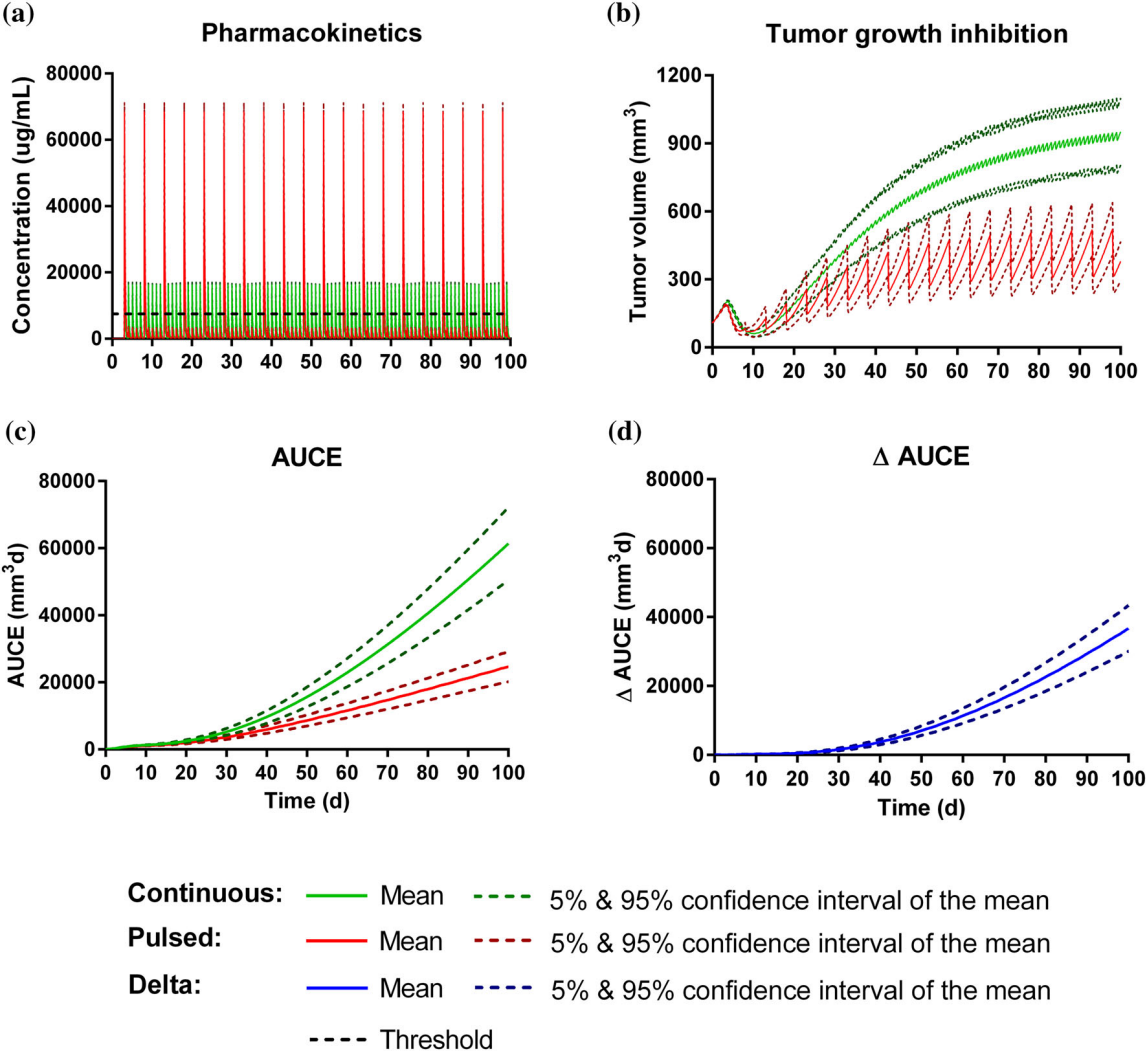
Simulation study with continuous versus pulsed dosing regimen of erlotinib in mice assuming a threshold concentration of 7152 μg/mL (black dashed) before affecting resistant cells, **a** PK of continuous, 100 mg/kg daily (green) and pulsed, 1 × 420 mg/kg and 4 × 20 mg/kg (red) dosing regimen, **b** corresponding tumor growth inhibition profiles shown as mean and 5 and 95% confidence interval of the mean, **c** the calculated AUCEs under continuous and pulsed treatment and **d** the difference in AUCE under continuous and pulsed treatment

We compared in silico resistance to drug treatment with continuous versus pulsed dosing regimen. Similar PD responses were obtained with the resistance model for both dosing regimens (data not shown). However, Chmielecki and colleagues [20] reported killing of resistant cells with in vitro at concentration above 1 μM of erlotinib. Similarly, for simulation purpose, the resistance model was modified and a treatment effect on resistant cells in vivo was assumed if free drug exposure exceeds the threshold concentration of 1 μM of erlotinib (MW = 393.4 g/mol) which corresponds to a total plasma threshold concentration (*Cth*) of 7150 μg/mL when correcting for fraction unbound of 0.055 [35]. In our experimental setting such effect on resistant cells could not be identified during model parameter estimation. With the modified resistance model (Supplementary material, S4), we compared in silico continuous with pulsed dosing regimen achieving the same average concentration in one treatment cycle. In line with our TGI studies, high dose was simulated for the continuous treatment with 100 mg/kg daily doses. The pulsed treatment cycle started with 420 mg/kg given on the first day, followed by 4 × daily administration of 20 mg/kg the following days. Simulated PK profiles of continuous and pulsed dosing regimen in mice are displayed in Fig. 7a. In both cases, the exposure exceeded the threshold concentration (Fig. 7a). With continuous dosing, concentration was maintained twofold above the threshold concentration during the treatment cycle whereas maximal plasma concentration exceeded the threshold concentration fourfold while exposure remained below the threshold concentration on 4 days of a 5 day cycle. When comparing the predicted tumor growth profiles (Fig. 7b) a better anti-tumor response was achieved with the pulsed dosing regimen (Fig. 7b). Figure 7c depicts the tumor burden over time under the two treatment schedules and Fig. 7d the difference between the two. It demonstrates a notably reduced tumor burden over the simulated time span with the pulsed treatment schedule. Other metrics, minimal tumor volume, time to tumor progression and difference in tumor burden after 30 and after 60 days under the two dosing regimen were calculated (Table 3). While minimal tumor volume and time to progression appeared similar for the two treatment schedules, the tumor burden over time (AUCE) was considerably lower under the pulsed treatment schedule.

**Table 3:** Comparison of continuous and pulsed treatment schedule in mice—derived metrics

Metric	Continuous	Pulsed
TV_min_ (mm^3^)	46.8 < **60.9** < 73.9	45.3 < **69.5** < 84.0
Time to progression (d)	14.8 < **16.9** < 19.9	13.2 < **14.8** < 23.7
ΔAUCE_30_	1231 < **1589** < 1947	
ΔAUCE_60_	9203 < **11357** < 13511	

TV_min_ is the minimal tumor volume and ΔAUCE the area under the curve of the tumor growth inhibition profile after 30 and 60 days respectively. The metrics for both dosing schedules are reported as mean (bold) and 5/95% confidence intervals of the mean

### Simulation studies to select dosing regimen for clinical trials with erlotinib

**Fig. 8:**
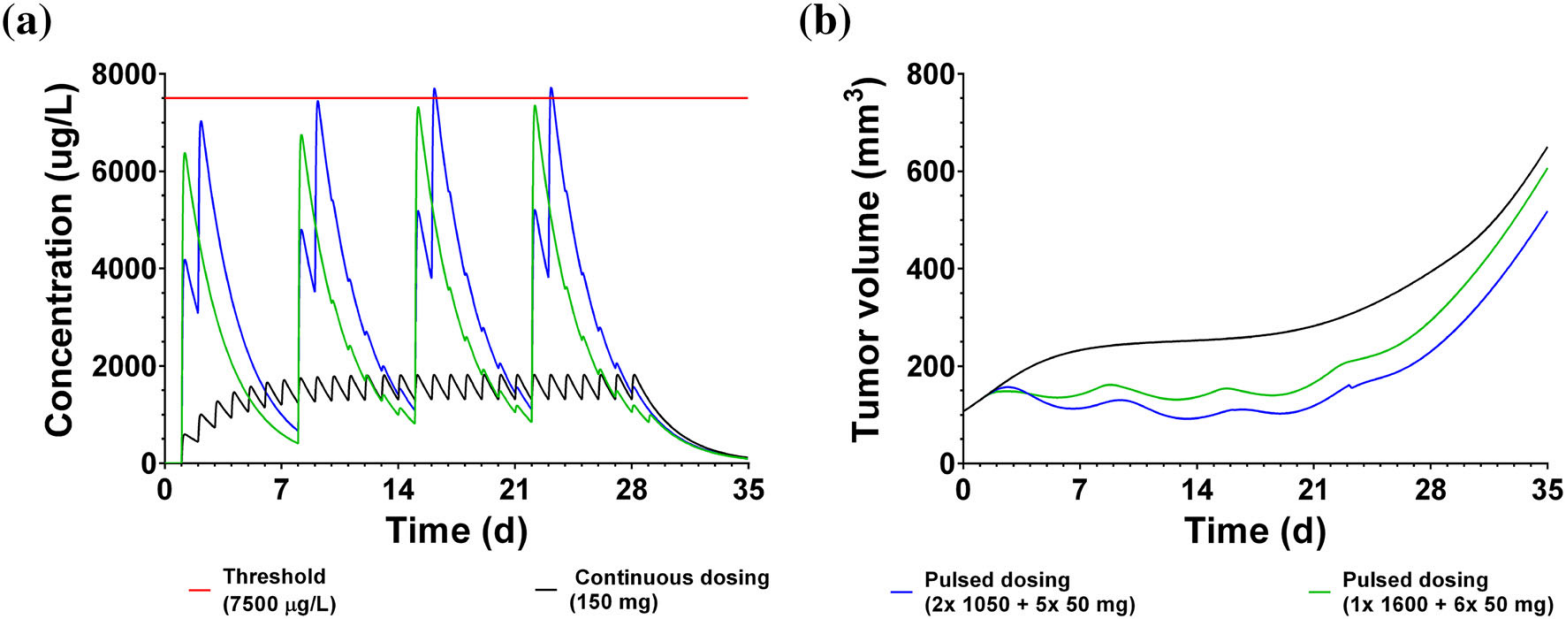
Simulation of clinical trial with erlotinib based on resistance model parameter estimation and with PK parameters of mice replaced by human parameter values. Simulation shows expected PK (**a**) and corresponding tumor growth behavior (**b**). Simulations are done for a continuous (black) and a pulsed (blue) treatment schedule. Different threshold concentrations to achieve an effect on resistant cells are simulated (red), with threshold 1 (solid red line) representing the value derived from published in previously published in vitro data and threshold 2 (dashed red line) representation more optimistic scenario. The predicted anti-tumor response is shown in (**b**) with continuous dose (solid black line), pulsed treatment assuming optimistic scenario (dashed blue line, threshold 2) or more pessimistic scenario (solid blue line)

Next, we explored in silico the impact of dosing regimen to reduce emergence of resistance in clinical trials with erlotinib. Standard continuous therapy (150 mg daily dosing) was compared to pulsed dosing regimen previously tested in clinical trials [37], namely two daily pulses of 1050 mg followed by 5 daily doses of 50 mg in 7 day cycle. Therefore, the mice PK parameters were replaced by human PK parameters (model code in supplementary material, S5). The simulated human PK profiles are shown in Fig. 8a. With regards to drug-induced killing of resistant cells, resistant cells will be killed if drug concentration is above 7150 μg/mL. With continuous dosing regimen of 150 mg daily doses, the exposure remains below this threshold concentration. With the pulsed treatment only very negligible exposure above threshold seems feasible. The clinical outcome was simulated (Fig. 8b) based on the hybrid PKPD model with human PK as an input to the acquired resistance model recapitulating the dynamics of emergence of resistance in mouse. This approach is limited when predicting the precise dynamic events of the clinical outcome, however it allows a relative comparison of various dosing regimen assuming that the relative differences in growth kinetics in mouse xenografts are likely preserved in cancer patients [24]. In summary, the simulation predicts a very minor potential delay of ~ 3 days under pulsed treatment relative to continuous dosing regimen.

## Discussion

NSCLC are heterogeneous tumors composed of multiple subclones [8]. Resistance may occur as a result of selection pressure mediated via the EGFR inhibition and clones with either intrinsic or acquired resistance are selected driving disease progression [8]. Emergence of acquired resistance has been reported in most patients with EGFR mutant NSCLC after long treatment with EGFR-TKI [9].

In the present study, we have investigated acquired resistance to treatment with erlotinib or gefitinib in patient-derived NSCLC xenografts to test if acquired resistance can be observed in preclinical studies and to simulate the dynamics of emergent resistance. Interestingly, resistance to drug treatment could only be detected by modeling and was not obvious from data exploration. An empirical nonlinear tumor growth model [32] was used to describe the unperturbed tumor growth kinetics. This is an important prerequisite to well characterize and quantify the drug effect [38], which is delayed relative to drug exposure in plasma. Based on our previous results [31], we considered plasma concentration as more relevant for the pharmacodynamic effects as compared to total tumor concentration. Erlotinib and gefitinib have different disposition profiles with gefitinib displaying a 21-fold higher tumor to plasma ratio of total drug concentration [31]. This is explained by the physicochemical properties of gefitinib, particularly the higher lipophilicity which is associated with a higher tissue partition. In addition, due to its pKa of 5.4 and 7.2 as higher binding in tumor tissue is expected due to ionic trapping in the acidic tumor microenvironment. These data suggested that tumor to plasma ratio which is based on total tumor concentration is not directly associated with a higher concentration of pharmacologically active drug.

The proposed model of acquired resistance is an extension of the base TGI model. It recapitalizes key processes of acquired resistance, which is defined by tumor-regrowth after initial tumor regression while patient is still under treatment [9] and assumes that drug treatment affects tumor cells sensitive to drug treatment which undergo several stages of damage before they eventually die or get converted to drug-resistant cells. This model requires only two additional parameters; *β*, denoting the ratio of growth rates of resistant versus sensitive cells and *kSR,* representing a rate which converts damaged tumor cells into resistant cells which are no longer killed by the drug and which have the ability to proliferate [39]. The base TGI and the acquired resistance model were both fitted to the combined dataset including control, gefitinib and erlotinib treatment groups. Based on parsimonious principles, improved model performance was demonstrated with the acquired resistance model obtaining a lower Akaike criterion, lower residual errors and improved goodness of fit plots. Furthermore, all parameters were well identified from the data set. One set of parameters was estimated for both drugs except for drug effect *k2*, which was drug-specific. The proposed model uses a three compartment transit model to capture the drug effect on tumor growth which is delayed relative to drug treatment [33]. This model assumes that upon drug action, a fraction of the tumor cells stop proliferating and proceed through progressive degree of damage (T1–T3) before they eventually die or become resistant. The secondary parameter *τ*, which denotes the average time it takes for tumor cell eradication or occurrence of resistance was calculated to be 1.97 days for both drugs. The assumption that the time delay between drug exposure and observed PD effect recapitulated by the transit rate parameter *k1* is the same for both drugs is substantiated with reports on experimental data demonstrating the similarity in molecular specificity particularly with regards to downstream signaling. Both, erlotinib and gefitinib are competitive inhibitors of the EGFR tyrosine kinase and have been shown to possess almost identical pharmacodynamic activities across a range of in vitro and xenograft experiments [31, 40]. Furthermore, it has been described, that the course of acquired resistance [20] as well as the molecular mechanisms [17] are the same for both drugs. Therefore, we assume that the parameters related to acquired resistance are the same.

We identified the high dose group as covariate on the conversion rate *kSR*. Interestingly, the concentration reached at this dose is above the threshold concentration above which cells are assumed to be killed when taking the in vitro results into consideration [21]. In addition, the high dose of erlotinib was identified as a covariate on the drug effect parameter *k2*, suggestion a higher potency of high dose group. This dose-dependency indicates a nonlinear drug effect relationship. A sigmoid Emax-model was tested to capture the nonlinear drug effect but underlying parameters could not be identified under the current study design. Furthermore, the parameters Emax and EC50 were highly correlated. This suggests that the dose range tested was not sufficient to explore the maximal response. The higher k2 value of the high dose can be interpreted as an increase in steepness of the dose–response curve at this dose level. This is most likely around the EC50 whereas the mid and low dose most likely below 20% of the maximal response. Measuring additional dose levels higher than 100 mg/kg or between the mid and high dose would allow exploring the non-linear drug effect. Another limitation of the current study design was that tumor samples were not tested for presence of resistant cells and the model-derived hypothesis was not substantiated with biological evidence. Genotyping of patient derived tumors in mice is recommended in further tumor growth inhibition studies to assess genetic alteration leading to TKI drug resistance.

Wang and colleagues reported that ~ 20–30% of NSCLC patients have no objective tumor regression on initial EGFR TKI treatment due to intrinsic or primary resistance to EGFR TKIs [39]. The proposed model does not capture primary resistance to drug treatment and is restricted to acquired resistance which occurs as a result of drug treatment. It should be noted that in the current study, initially, all animals responded to the drug treatment. In addition, it was not possible to estimate an initial pool of resistant cells (data not shown) to the observed data most likely because resistant tumor cells are negligible at the start of the study [21]. The model was applied to assess the dynamic processes of acquired resistance which is of clinical relevance. We simulated the response on overall tumor growth and the accumulation of resistant cells during and after long term treatment assuming no drug effect on resistant cells. The model predicts tumor shrinkage at the beginning of drug treatment followed by relapse during treatment with a high fraction of resistant cells. In line with clinical reports, the model suggests a worsening of diseases progression after discontinuation of drug treatment due to the rebound of faster growing sensitive tumor cells [10, 15, 20]. This explanation is further supported by in vitro findings [21] demonstrating that that sensitive and drug resistant EGFR-mutant cells exhibit differential growth dynamics with drug resistant cells showing slower growth. Furthermore, resistant tumors are composed of a heterogeneous mix of TKI-sensitive and -resistant tumor cells and that stopping TKI therapy may permit expansion [21]. In this in vitro study, EGFR-resistant cells were killed by high concentration of erlotinib and it was suggested that a pulsed treatment with high doses followed by lower doses might be more beneficial than the traditional continuous dosing schedule. Interestingly, a clinical trial was initiated in order to test this potential benefit [37]. In the present study, we could not identify a killing term on resistant cells (data not shown). However, in simulations studies we predicted a reduction in overall tumor volume when simulation dosing schedules achieving exposures above the threshold concentration of 7150 μg/mL above which resistant cells is assumed to be killed. With this model expansion, we explored, based on in silico simulations, if the suggested pulsed dosing regimen is superior to continuous treatment. In mice, we compared continuous to pulsed dosing regimen with both regimen achieving the same average concentration. After long term treatment, the pulsed dosing regimen is predicted to have an improved anti-tumor response in mice albeit only short exposure peaks above the threshold concentration are achieved. The continuous dosing regimen did not prevent emergence of resistance despite having exposure levels slightly above the threshold during the whole treatment period. Based on PK data only, this result may be counterintuitive and illustrates how in silico exploration can be used to better understand the driving forces of a PD endpoint. In a next step, it is proposed to test this new hypothesis experimentally and to conduct vivo experiments to verify the outcome of the simulation study. This will increase the confidence that intermittent dosing regimen has an impact and reduces emergence of resistance.

The proposed PKPD model is a semi-mechanistic model which allows mechanistic interpretation of the drug effect and enables simulations of untested scenarios. Several retrospective studies could show a clear relationship of the predicted efficacious exposure derived from the translational PKPD model and the observed clinical activity in cancer patients when accounting for the PK differences between the species [31, 40, 41]. Therefore, we proposed a hybrid PKPD model where human PK is used as input to the acquired resistance model developed in mice. Tumor growth rate in patient is generally observed slower than in mice and the proposed hybrid PKPD model is not intended to predict tumor growth dynamic in treated patients. Killing rate is also described as correlated to tumor growth rate [42] and one could assume that net effect (growth minus killing) could indeed translate from mice to human without parameter scaling. This approach is supported by published translational PKPD models [31, 40, 41]. If the goal was to predict the time course of tumor growth and shrinkage in patients, then all PKPD model components need to be scaled to cancer patients. The challenge is to obtain relevant parameters on tumor growth and shrinkage which greatly depends on the context. For instance, it is reported that the doubling time of cancer cells in in vitro systems is much faster as compared to the doubling time after engrafting into xenograft mice [43]. In addition, the timescale of tumor shrinkage in mice greatly differs cancer patients [42]. In summary, when scaling the full PKPD model to humans, additional uncertainty is introduced which will reduced the confidence in model prediction. Therefore, the hybrid model was seen as the better approach to compare different dosing regimens without the additional burden of scaling tumor growth and tumor shrinkage parameters.

With the hybrid model, we simulated the expected response in cancer patients of a previously reported improved treatment schedule based on an evolutionary modeling approach [21, 44]. This dosing schedule combines high-dose pulse and daily low-dose administration of erlotinib and was previously tested in a Phase I trial [37]. The results of this Phase I study showed no improvement with regards to progression free survival or delay of emergence of resistance [37]. In line, our simulation results showed a minor delay in tumor-regrowth compared to the standard therapy treatment of 150 mg daily doses. It was concluded that the pulse dosing was too low to affect resistant cells to ultimately improve the clinical outcome. Higher doses would need to be administered in order to achieve sufficient exposure to kill resistant cells. However, this will be limited to the tolerability of high doses. In order to increase confidence in this threshold concentration, it is suggested to compare this experimentally as proposed with the simulation study which suggests a benefit of the pulsed dosing regimen. In this study, continuous and pulsed dosing regimen are compared with regards to tumor growth. According to the simulations, this beneficial effect could become apparent after 60 days of treatment. The proposed dosing regimen will achieved the same overall exposure. The continuous dosing regimen remains below the threshold concentration, whereas the pulsed treatment reaching concentration above the threshold concentration.

## Conclusion

We proposed a semi-mechanistic model following parsimonious principles to characterize emergence of resistance to drug treatment in patient derived NSCLC xenograft mice. The model provides mechanistic insights into the dynamic processes of acquired resistance. It predicts tumor regrowth during treatment driven by the selection of resistant cells and it offers an explanation why faster tumor regrowth may occur after discontinuation of TKI treatment. The model was further applied to compare in silico different dosing regimen and their impact on acquired resistance. This allows to explore hypothesis and to design studies allowing testing those. Finally, we propose to explore in silico different scenarios and to identify optimal treatment schedules for clinical trials.

## Electronic supplementary material

Below is the link to the electronic supplementary material.

Supplementary material 1 (DOCX 386 kb)
