# Supplementary material for: PKPD modeling of acquired resistance to anti-cancer drug treatment
Source: J Pharmacokinet Pharmacodyn. 2017 Oct 31;44(6):617–30. doi: 10.1007/s10928-017-9553-x (PMC5686279; doi:10.1007/s10928-017-9553-x)
Supplement: Supplementary file 1 — Supplementary material 1 (DOCX 386 kb) [file 10928_2017_9553_MOESM1_ESM.docx]

**Supplementary material: PKPD modeling of acquired resistance to anti-cancer drug treatment**

*Journal of Pharmacokinetics and Pharmacodynamics*

Miro J. Eigenmann^1^, Nicolas Frances^1^, Thierry Lavé^2^, Antje-Christine Walz^1^

**Authors’ Affiliations:** Roche Pharma Research and Early Development, ^1^Quantitative Systems Pharmacology, ^2^Project leaders and M&S, Pharmaceutical Sciences Roche Innovation Centre Basel, Hoffmann-La Roche Ltd., Basel

**Email corresponding author:** [antje-christine.walz@roche.com](mailto:antje-christine.walz@roche.com)

**Supplementary Table S1** Experimental design of the in vivo study in tumor bearing mice. Due to dose-limiting toxicity in the high dose group with erlotinib, a drug holiday was introduced

| Treatment | erlotinib | gefitinib |
| --- | --- | --- |
| Tumor type | Patient derived NSCLC LXFA677 | |
| Dose groups (mg/kg) | control: 0  low dose: 6.25  mid dose: 25  high dose 100 | |
| Animals/group | 8 | |
| Study duration | 30 days | |
| Dosing days | low & mid dose: Day 3-16  high dose: Day 3-10; 14-16 | Day 3-16 |
| PK sampling days | Day 10 and Day 16 |  |
| PK sampling | 1 sample/time point/dose group  2 samples/animal | |
| Tumor volume measurement | every 2-3 days | |

**Supplementary Table S2** Summary of missing TGI data in the various treatment groups. E indicates erlotinib and G stands for gefitinib. Total number of missing samples, number of affected animals and the reason for the missing data is indicated in the different columns. Missing data samples as percentage of total number of samples is reported in the bottom row.

| **Treatment group** |  | **# Missing data** |  | **# Below quantification** |  | **# Truncated data** |  | **# affected animals** | | |  |
| --- | --- | --- | --- | --- | --- | --- | --- | --- | --- | --- | --- |
| Vehicle |  | 10/112 |  | 0 |  | 10 | | |  | 3/8 |  |
| E 6.25 |  | 5/112 |  | 0 |  | 5 | | |  | 2/8 |  |
| E 25 |  | 0/112 |  | 0 |  | 0 | | |  | 0/8 |  |
| E 100 |  | 9/112 |  | 1 |  | 8 | | |  | 2/8 |  |
| G 6.25 |  | 6/112 |  | 0 |  | 6 | | |  | 3/8 |  |
| G 25 |  | 7/112 |  | 0 |  | 7 | | |  | 3/8 |  |
| G 100 |  | 15/112 |  | 6 |  | 9 | | |  | 5/8 |  |
| % total |  | 7% |  | 0.9% |  | 6% | | |  | 32% |  |

**S3, Berkeley Madonna code: Long term treatment in mice to simulate dynamic events of emergence resistance**

METHOD STIFF

STARTTIME = 0

STOPTIME=50

DT = 0.02

TOLERANCE = 0.000001

;Parameters

;---------------------------------------------------------------------------------

KA=55 ; Absorption rate (1/d)

V=0.127 ; Volume (L)

KE=7.56 ; Elimination rate (1/d)

l0S=0.217 ; Exponential growth rate, sensitive cells (mm^3/d)

beta=0.869 ; Ratio resistant/sensitive growth

l1S=42.8 ; Linear growth rate, sensitive cells (1/d)

l1R=l1S*beta ; Linear growth rate, resistant cells (1/d)

l0R=l0S * beta ; Exponential growth rate, resistant cells (mm^3/d)

TV0=107 ; Initial tumor volume (mm^3)

kSR=0.0074 ; Transformation rate from sensitive to resistant cells (1/d)

kSR_hd = 0.00166 ; Transformation rate from sensitive to resistant cells (1/d) upon high dose

k1 = 1.51 ; Delay between PK and drug effect (d)

k2=0.0000921 ; Kill rate sensitive cells (ug/L)

k2_hd=0.000315 ; Kill rate sensitive cells (ug/L) high dose

FR=R/TV ; Fraction resistant cells

;ODE

;---------------------------------------------------------------------------------

;PK

;---------------------------------------------------------------------------------

A1' = Input - KA*A1 ; Amount drug in depot compartment (ug)

A2' = KA * A1 - ( KE ) * A2 ; Amount drug in plasma (ug)

PK=A2/V ; Conversion to plasma concentration (ug/L)

AUC'=PK ; Cumulative exposure

;PKPD

;---------------------------------------------------------------------------------

TV= S+T1+T2+T3+R ; Tumor volume (mm^3)

S' = 2 * l0S * l1S * S / ( l1S + 2 * l0S * S ) - k2_hd * S * PK ; Sensitive cells (mm^3)

T1'= k2_hd*S*PK - T1 * k1 ; Damaged cells in transit compartment 1 (mm^3)

T2'= k1 * (T1-T2) ; Damaged cells in transit compartment 2 (mm^3)

T3'= k1 * (T2-T3) - T3*kSR ; Damaged cells in transit compartment 3 (mm^3)

R' = T3 * kSR + 2 * l0R *l1R* R /(2*l0R * R + l1R) ; Resistant cells (mm^3)

;Intial conditions

;--------------------------------------------------------------------------------

INIT(A1)=0

INIT(A2)=0

INIT(T1)=0

INIT(T2)=0

INIT(T3)=0

INIT(S)= TV0 ; initial tumor volume, sensitive cells

INIT(R)=0 ; initial tumor volume, resistant cells

INIT(AUC)=0

;Dosing

;----------------------------------------------------------------------

;Continuous Treatment

;----------------------------------------------------------------------

start1=3 ; Start of treatment period 1

dose_n=100 ; Normalized dose (mg/kg)

C=25 ; Conversion to flat dose in ug

dose1=dose_n*C ; Flat dose per day (ug)

n1=20 ; Total number of doses to be administered (period 1)

interval1=1 ; Time interval between doses (d)

Input=pulse(dose1, start1, interval1) - pulse(dose1, start1+n1*interval1, interval1)

Display TV, PK, S, R, FR

**S4, Berkeley Madonna code: Continuous versus pulsed dose in xenograft mice assuming killing of resistant cells at high concentration**

METHOD STIFF

STARTTIME = 0

STOPTIME=100

DT = 0.1

TOLERANCE = 0.000001

nsim=250

; Fixed effect parameters

;---------------------------------------------------------------------------------

KA=55 ; Absorption rate (1/d)

V2=0.127 ; Volume (L)

KE=7.56 ; Elimination rate (1/d)

l0S=0.217 ; Exponential growth rate, sensitive cells (mm^3/d)

beta=0.869 ; Ratio resistant/sensitive growth

l1S=42.8 ; Linear growth rate, sensitive cells (1/d)

l1R[1..nsim]=l1S_ind[i] * beta_ind[i] ; Linear growth rate, resistant cells (1/d)

l0R[1..nsim]=l0S_ind[i] * beta_ind[i] ; Exponential growth rate, resistant cells (mm^3/d)

w0=107 ; Initial tumor volume (mm^3)

kSR_ld=0.0074 ; Transformation rate from sensitive resistant cells (1/d) low dose

kSR_hd=0.00166 ; Transformation rate from sensitive resistant cells (1/d) high dose

k1=1.51 ; Delay between PK and drug effect (d)

k2_ld=0.0000921 ; Kill rate sensitive cells (ug/L) low dose

k2_hd=0.000315 ; Kill rate sensitive cells (ug/L) high dose

FR[1..nsim]=R[i]/TV[i] ; Fraction resistant cells

Cth= 7152 ; Derived from Chmielecki et al. 2011 (in vitro) & corrected for free fraction

;Random effect of model parameters

;---------------------------------------------------------------------------------

eta_KA=0 ; Absorption rate (1/d)

eta_V2=0.251 ; Volume (L)

eta_KE=0.332 ; Elimination rate (1/d)

eta_l0S=0.543 ; Exponential growth rate, sensitive cells (mm^3/d)

eta_beta=0.742 ; Ratio resistant/sensitive growth

eta_l1S=0.65 ; Linear growth rate, sensitive cells (1/d)

eta_kSR_ld=0.1 ; Transformation rate from sensitive resistant cells (1/d) low dose

eta_kSR_hd=0.1 ; Transformation rate from sensitive resistant cells (1/d) high dose

eta_k1=0.2 ; Delay between PK and drug effect (d)

eta_k2_ld=0.437 ; Kill rate sensitive cells (ug/L) low dose

eta_k2_hd=0.437 ; Kill rate sensitive cells (ug/L) high dose

;Vector of individual parameters

;---------------------------------------------------------------------------------

init eta_KA_ind[1..nsim] = normal(0, eta_KA)

next eta_KA_ind[1..nsim] = eta_KA_ind[i]

KA_ind[1..nsim]=KA*exp(eta_KA_ind[i])

init eta_V2_ind[1..nsim] = normal(0, eta_V2)

next eta_V2_ind[1..nsim] = eta_V2_ind[i]

V2_ind[1..nsim]=V2*exp(eta_V2_ind[i])

init eta_KE_ind[1..nsim] = normal(0, eta_KE)

next eta_KE_ind[1..nsim] = eta_KE_ind[i]

KE_ind[1..nsim]=KE*exp(eta_KE_ind[i])

init eta_l0S_ind[1..nsim] = normal(0, eta_l0S)

next eta_l0S_ind[1..nsim] = eta_l0S_ind[i]

l0S_ind[1..nsim]=l0S*exp(eta_l0S_ind[i])

init eta_l1S_ind[1..nsim] = normal(0, eta_l1S)

next eta_l1S_ind[1..nsim] = eta_l1S_ind[i]

l1S_ind[1..nsim]=l1S*exp(eta_l1S_ind[i])

init eta_beta_ind[1..nsim] = normal(0, eta_beta)

next eta_beta_ind[1..nsim] = eta_beta_ind[i]

beta_ind[1..nsim]=beta*exp(eta_beta_ind[i])

init eta_kSR_ld_ind[1..nsim] = normal(0, eta_kSR_ld)

next eta_kSR_ld_ind[1..nsim] = eta_kSR_ld_ind[i]

kSR_ld_ind[1..nsim]=kSR_ld*exp(eta_kSR_ld_ind[i])

init eta_kSR_hd_ind[1..nsim] = normal(0, eta_kSR_hd)

next eta_kSR_hd_ind[1..nsim] = eta_kSR_hd_ind[i]

kSR_hd_ind[1..nsim]=kSR_hd*exp(eta_kSR_hd_ind[i])

init eta_k1_ind[1..nsim] = normal(0, eta_k1)

next eta_ k1_ind[1..nsim] = eta_ k1_ind[i]

k1_ind[1..nsim]= k1*exp(eta_ k1_ind[i])

init eta_k2_ld_ind[1..nsim] = normal(0, eta_k2_ld)

next eta_k2_ld_ind[1..nsim] = eta_k2_ld_ind[i]

k2_ld_ind[1..nsim]=k2_ld*exp(eta_k2_ld_ind[i])

init eta_k2_hd_ind[1..nsim] = normal(0, eta_k2_hd)

next eta_k2_hd_ind[1..nsim] = eta_k2_hd_ind[i]

k2_hd_ind[1..nsim]=k2_hd*exp(eta_k2_hd_ind[i])

k2R[1..nsim]= IF (PK[i]>Cth) THEN 0.0000921 ELSE 0 ; Effect on resistant cells upon reaching the threshold concentration

k2R_pulse[1..nsim]= IF (PK_pulse[i]>Cth) THEN 0.0000921 ELSE 0

k2[1..nsim] = IF(PK[i] < 7000) THEN k2_ld ELSE k2_hd

kSR[1..nsim] = IF(PK[i] < 7000) THEN kSR_ld ELSE kSR_hd

;ODE

;=========================================

;PK

;=========================================

;PK for continuous treatment

;----------------------------------------

A1'[1..nsim] = Input - KA_ind[i]*A1[i] ; Amount drug in depot department

A2'[1..nsim] = KA_ind[i] * A1[i] - ( KE_ind[i] ) * A2[i] ; Amount drug in plasma

PK[1..nsim]=A2[i]/V2_ind[i] ; Conversion to plasma concentration

AUC'[1..nsim]=PK[i] ; Cumulative exposure

;PK for pulsed treatment

;----------------------------------

B1'[1..nsim] = Input2 - KA_ind[i]*B1[i]

B2'[1..nsim] = KA_ind[i] * B1[i] - ( KE_ind[i] ) * B2[i]

PK_pulse[1..nsim]=B2[i]/V2_ind[i]

AUC_pulse'[1..nsim]=PK_pulse[i]

;PKPD

;=========================================

;PKPD for continuous treatment

;---------------------------------------------

TV[1..nsim]= S[i]+T1[i]+T2[i]+T3[i]+R[i] ; Tumor volume

S'[1..nsim] = 2 * l0S_ind[i] * l1S_ind[i] * S[i] / ( l1S_ind[i] + 2 * l0S_ind[i] * S[i] ) - k2_hd_ind[i] * S[i] * PK[i] ; Sensitive cells

T1'[1..nsim]= k2_hd_ind[i]*S[i]*PK[i] - T1[i] * k1_ind[i] ; Damaged cells in transit compartment 1

T2'[1..nsim]= k1_ind[i] * (T1[i]-T2[i]) ; Damaged cells in transit compartment 2

T3'[1..nsim]= k1_ind[i] * (T2[i]-T3[i]) - T3[i]*kSR_hd_ind[i] ; Damaged cells in transit compartment 3

R'[1..nsim] = T3[i] * kSR_hd_ind[i] + 2 * l0R[i] *l1R[i]* R[i] /(2*l0R[i] * R[i] + l1R[i]) - R[i]*k2R[i]*(PK[i]-Cth) ; Resistant cells

AUCE'[1..nsim]=TV[i]

;PKPD for pulsed treatment

;---------------------------------------

TV_pulse[1..nsim]= S_pulse[i]+T1_pulse[i]+T2_pulse[i]+T3_pulse[i]+R_pulse[i]

S_pulse'[1..nsim] = 2 * l0S_ind[i] * l1S_ind[i] * S_pulse[i] / ( l1S_ind[i] + 2 * l0S_ind[i] * S_pulse[i] ) - S_pulse[i]* kSR_hd_ind[i] - k2_hd_ind[i] * S_pulse[i] * PK_pulse[i]

T1_pulse'[1..nsim]= k2_hd_ind[i]*S_pulse[i]*PK_pulse[i] - T1_pulse[i] * k1_ind[i]

T2_pulse'[1..nsim]= k1_ind[i] * (T1_pulse[i]-T2_pulse[i])

T3_pulse'[1..nsim]= k1_ind[i] * (T2_pulse[i]-T3_pulse[i]) - T3_pulse[i]*kSR_hd_ind[i]

R_pulse'[1..nsim] = T3_pulse[i] * kSR_hd_ind[i] + 2 * l0R[i] *l1R[i]* R_pulse[i] /(2*l0R[i] * R_pulse[i] + l1R[i]) - R_pulse[i]*k2R_pulse[i]*(PK_pulse[i]-Cth)

AUCE_pulse'[1..nsim]=TV_pulse[i]

Threshold = Cth

deltaAUCE[1..nsim]=AUCE[i]-AUCE_pulse[i]

;Intial conditions

;--------------------------------------------------------------------------------

INIT(A1[1..nsim])=0

INIT(A2[1..nsim])=0

INIT(T1[1..nsim])=0

INIT(T2[1..nsim])=0

INIT(T3[1..nsim])=0

INIT(S[1..nsim])= w0

INIT(R[1..nsim])=0

INIT(AUC[1..nsim])=0

INIT(AUCE[1..nsim])=0

INIT(B1[1..nsim])=0

INIT(B2[1..nsim])=0

INIT(T1_pulse[1..nsim])=0

INIT(T2_pulse[1..nsim])=0

INIT(T3_pulse[1..nsim])=0

INIT(S_pulse[1..nsim])= w0 ; initial tumor volume, sensitive cells

INIT(R_pulse[1..nsim])=0 ; initial tumor volume, resistant cells

INIT(AUC_pulse[1..nsim])=0

INIT(AUCE_pulse[1..nsim])=0

;Dosing

;----------------------------------------------------------------------

;Continuous Treatment

;----------------------------------------------------------------------

start1=3 ; Start of treatment period 1

dose_n=100 ; Normalized dose (mg/kg)

C=25 ; Conversion to flat dose in ug

dose1=dose_n*C ; Flat dose per day (ug)

n1=100 ; Total number of doses to be administered

interval1=1 ; Time interval between doses (d)

Input=pulse(dose1, start1, interval1) - pulse(dose1, start1+n1*interval1, interval1)

;Pulsed Treatment

;----------------------------------------------------------------------

start1=3 ; Start of treatment period 1

n1=100 ; Total number of doses to be administered

interval2=5 ; Time interval between pulsed doses (d)

dose_n2=400 ; Normalized dose pulse (mg/kg)

dose_n3=20 ; Normalized dose following pulse (mg/kg)

dose2=dose_n2*C ; Pulse dose (ug) (Added on continuous low dose)

dose3=dose_n3*C ; Low dose following pulse (ug)

interval3=1 ; Time interval between continuous doses (d)

n2=100

Input2 = pulse(dose2, start1, interval2) - pulse(dose2, start1+n1*interval2, interval2) + pulse(dose3, start1, interval3) - pulse(dose3, start1+n2*interval3, interval3)

Display PK, PK_pulse, TV, TV_pulse, AUCE, AUCE_pulse, deltaAUCE

Display kSR_hd, k2_hd, tau, Cth

**S5, Berkeley Madonna code: Simulations of clinical trial in cancer patients. Continuous versus pulsed dosing assuming killing of resistant cells at high concentration**

METHOD STIFF

STARTTIME = 0

STOPTIME=60

DT = 0.02

TOLERANCE = 0.000001

;Parameters

;============================================================================

;Human PK Parameters

;Literature values from Lu et al., Clinical Pharmacology & Therapeutics, 2006;80(2):136-45)

;============================================================================

KA = 22.8 ; absorption rate (1/d)

V = 233 ; Volume (L)

KE = 0.408 ; elimination rate (1/d)

;PD Parameters

;Taken from mouse estimates

;============================================================================

l0S=0.217 ; exponential growth rate, sensitive cells (mm^3/d)

beta=0.869 ; ratio resistant/sensitive growth

l1S=42.8 ; linear growth rate, sensitive cells (1/d)

l1R=l1S*beta ; linear growth rate, resistant cells (1/d)

l0R=l0S * beta ; exponential growth rate, resistant cells (mm^3/d)

TV0=107 ; initial tumor volume (mm^3)

kSR=0.00166 ; generation rate of resistant cells (1/d)

k1=1.51 ; delay between PK and drug effect (d)

k2=0.0000921 ; kill rate sensitive cells (ug/L)

FR=R/TV ; Resistant fraction

Cth= 7152 ; Derived from Chmielecki et al. 2011 (in vitro) & corrected for free fraction

Threshold = Cth

k2R= IF (PK>Cth) THEN 0.0000921 ELSE 0 ;effect on resistant cells after reaching the threshold concentration

;ODE

;============================================================================

;PK

;============================================================================

;PK for continuous treatment

;----------------------------------------

A1' = Input_c - KA*A1 ;amount drug in depot compartment

A2' = KA * A1 - ( KE ) * A2 ;amount drug in plasma

PK=A2/V ;conversion from to plasma concentration

AUC'=PK ;cumulative exposure

;PK for pulsed treatment

;----------------------------------

E1' = Input_p4 - KA*E1

E2' = KA * E1 - ( KE ) * E2

PK_p4=E2/V

AUC_p4'=PK_p4

;PK for pulsed treatment 2

;-------------------------------------

C1' = Input_p - KA*C1

C2' = KA * C1 - ( KE ) * C2

PK_p=C2/V

AUC_p'=PK_p

;PKPD

;============================================================================

;PKPD continuous

;-------------------------

TV= S+T1+T2+T3+R ;tumor volume (mm^3)

S' = 2 * l0S * l1S * S / ( l1S + 2 * l0S * S ) - k2* S * PK ;sensitive cell population (mm^3)

T1'= k2*S*PK - T1 * k1 ;damaged cells in transit compartment 1

T2'= k1 * (T1-T2) ;damaged cells in transit compartment 2

T3'= k1 * (T2-T3) - T3*kSR ;damaged cells in transit compartment 3

R' = T3 * kSR + 2 * l0R *l1R* R /(2*l0R * R + l1R) - k2R * PK * R ;resistant cell population (mm^3)

FR = R/TV

;PKPD for pulsed dosing

;-----------------------------------

k2R_p4= IF (PK_p4>Cth) THEN 0.0000921 ELSE 0

TV_p4= S_p4+T1_p4+T2_p4+T3_p4+R_p4

S_p4' = 2 * l0S * l1S * S_p4 / ( l1S + 2 * l0S * S_p4 ) - k2* S_p4 * PK_p4

T1_p4'= k2*S_p4*PK_p4 - T1_p4 * k1

T2_p4'= k1 * (T1_p4-T2_p4)

T3_p4'= k1 * (T2_p4-T3_p4) - T3_p4*kSR

R_p4' = T3_p4 * kSR + 2 * l0R *l1R* R_p4 /(2*l0R * R_p4 + l1R) - k2R_p4 * PK_p4 * R_p4

FR_p4 = R_p4/TV_p4

;PKPD for pulsed dosing 2

;--------------------------------------

k2R_p= IF (PK_p>Cth) THEN 0.0000921 ELSE 0

TV_p= S_p+T1_p+T2_p+T3_p+R_p

S_p' = 2 * l0S * l1S * S_p / ( l1S + 2 * l0S * S_p ) - k2* S_p * PK_p

T1_p'= k2*S_p*PK_p - T1_p * k1

T2_p'= k1 * (T1_p-T2_p)

T3_p'= k1 * (T2_p4-T3_p) - T3_p*kSR

R_p' = T3_p * kSR + 2 * l0R *l1R* R_p /(2*l0R * R_p + l1R) - k2R_p * PK_p * R_p

FR_p = R_p/TV_p

;Initial conditions

;============================================================================

;Continuous dosing 4

;-----------------------

INIT(A1)=0

INIT(A2)=0

INIT(T1)=0

INIT(T2)=0

INIT(T3)=0

INIT(S)= TV0 ; initial tumor volume, sensitive cells

INIT(R)=0 ; initial tumor volume, resistant cells

INIT(AUC)=0

;Pulsed dosing

;--------------------

INIT(E1)=0

INIT(E2)=0

INIT(T1_p4)=0

INIT(T2_p4)=0

INIT(T3_p4)=0

INIT(S_p4)= TV0 ; initial tumor volume, sensitive cells

INIT(R_p4)=0 ; initial tumor volume, resistant cells

INIT(AUC_p4)=0

;Pulsed dosing

;--------------------

INIT(C1)=0

INIT(C2)=0

INIT(T1_p)=0

INIT(T2_p)=0

INIT(T3_p)=0

INIT(S_p)= TV0 ; initial tumor volume, sensitive cells

INIT(R_p)=0 ; initial tumor volume, resistant cells

INIT(AUC_p)=0

;Implementing treatment schedule

;============================================================================

dose_c=150*1000 ;Clinically prescribed dose (150mg)

dose_l=50*1000 ;Low dose following pulse (50mg)

dose_p4=1050*1000 ;Pulsed dosing 4 (1050mg)

dose_p = 1600*1000

start1_c=1 ;Start of dosing (day)

start1_p=1 ;Start of pulsed dosing (day)

start2_p=start1_p+7 ;Time of second pulses (day)

start3_p=start2_p+7 ;Time of third pulses (day)

start4_p=start3_p+7 ;Time of fourth pulses (day)

start1_l=10 ;Start of low dose administration after initial pulses

start2_l=start1_l+7

start3_l=start2_l+7

interval1=1 ;Time between dosing (day)

n_c=28 ;Number of doses for continuous treatment

n_p=2 ;Number of pulsed doses in each sequence

n_p2=1

n_l=5 ;Number of low doses following pulses in each sequence, except 1st week

n_lp2=6

;Traditional continuous dosing with clinically prescribed dose

;============================================================================

Input_c=pulse(dose_c, start1_c, interval1) - pulse(dose_c, start1_c+n_c*interval1, interval1)

;Pulsed dosing

;============================================================================

Input_p4 = pulse(dose_p4, start1_p, interval1) - pulse(dose_p4, start1_p+n_p*interval1, interval1) +pulse(dose_p4, start2_p, interval1) - pulse(dose_p4, start2_p+n_p*interval1, interval1) + pulse(dose_p4, start3_p, interval1) - pulse(dose_p4, start3_p+n_p*interval1, interval1) + pulse(dose_p4, start4_p, interval1) - pulse(dose_p4, start4_p+n_p*interval1, interval1) + pulse(dose_l, start1_l, interval1) - pulse(dose_l, start1_l+n_l*interval1, interval1)+ pulse(dose_l, start2_l, interval1) - pulse(dose_l, start2_l+n_l*interval1, interval1)+ pulse(dose_l, start3_l, interval1) - pulse(dose_l, start3_l+n_l*interval1, interval1)

;Pulsed dosing 2

;============================================================================

Input_p = pulse(dose_p, start1_p, interval1) - pulse(dose_p, start1_p+n_p2*interval1, interval1) +pulse(dose_p, start2_p, interval1) - pulse(dose_p, start2_p+n_p2*interval1, interval1) + pulse(dose_p, start3_p, interval1) - pulse(dose_p, start3_p+n_p2*interval1, interval1) + pulse(dose_p, start4_p, interval1) - pulse(dose_p, start4_p+n_p2*interval1, interval1) + pulse(dose_l, start1_l, interval1) - pulse(dose_l, start1_l+n_lp2*interval1, interval1)+ pulse(dose_l, start2_l, interval1) - pulse(dose_l, start2_l+n_lp2*interval1, interval1)+ pulse(dose_l, start3_l, interval1) - pulse(dose_l, start3_l+n_lp2*interval1, interval1)

Display TV, TV_p4, TV_p, PK, PK_p, PK_p4, R, R_p, R_p4, S, S_p, S_p4, Threshold

Display Cth

**Supplementary Figure S6 – Individual TGI profiles of the high dose group**





**S6:** Shows all individual TGI profiles in the high dose (HD) group for erlotinib (E) and gefitinib (G). Individuals with apparent reduced drug treatment effect are titled in red. These individuals either show regrowth under treatment or a reduced tumor regression under treatment. The dosing schedule is indicated by the black triangles and measured data by the solid circles.

**Supplementary Table S7 – Ω correlation matrix**

| **Parameter** | λ_0_ | λ_1_ | k2 erlotinib | | k2 erlotinib high dose | k2 gefitinib | kSR | | kSR high dose | k1 | β |
| --- | --- | --- | --- | --- | --- | --- | --- | --- | --- | --- | --- |
| λ_0_ | 1 |  |  | |  |  |  | |  |  |  |
| λ_1_ | -0.28 | 1 |  | |  |  |  | |  |  |  |
| k2 erlotinib | 0.09 | 0.05 | 1 | |  |  |  | |  |  |  |
| k2 erlotinib  high dose | -0.02 | -0.04 | -0.8 | | 1 |  |  | |  |  |  |
| k2 gefitinib | 0.14 | -0.04 | -0.07 | | 0.08 | 1 |  | |  |  |  |
| kSR | -0.01 | -0.01 | 0.18 | | -0.16 | -0.06 | 1 | |  |  |  |
| kSR high dose | 0 | 0.07 | -0.04 | | 0.1 | 0.02 | -0.28 | | 1 |  |  |
| k1 | -0.13 | 0.11 | 0 | | -0.06 | -0.24 | 0.15 | | -0.12 | 1 |  |
| β | -0.09 | -0.07 | -0.06 | | -0.01 | -0.08 | 0.08 | | -0.14 | 0.01 | 1 |
| **Eigenvalues** | | | | | | | | | | | |
| **Min.** | | | | **Max.** | | | | **Max./Min.** | | | |
| 0.83 | | | | 1.2 | | | | 1.5 | | | |
